# Axial mitochondrial myopathy in a patient with rapidly progressive adult-onset scoliosis

**DOI:** 10.1186/s40478-014-0137-3

**Published:** 2014-09-16

**Authors:** Annie Hiniker, Lee-Jun Wong, Sigurd Berven, Cavatina K Truong, Adekunle M Adesina, Marta Margeta

**Affiliations:** 1 Department of Pathology, University of California San Francisco, San Francisco, CA USA; 2 Department of Molecular Human Genetics, Baylor College of Medicine, Houston, TX USA; 3 Department Orthopedic Surgery, University of California San Francisco, San Francisco, CA USA; 4 Department of Pathology and Immunology, Baylor College of Medicine, Houston, TX USA

**Keywords:** mtDNA, Mitochondrial myopathy, Camptocormia, Adult scoliosis

## Abstract

Axial myopathy can be the underlying cause of rapidly progressive adult-onset scoliosis; however, the pathogenesis of this disorder remains poorly understood. Here we present a case of a 69-year old woman with a family history of scoliosis affecting both her mother and her son, who over 4 years developed rapidly progressive scoliosis. The patient had a history of stable scoliosis since adolescence that worsened significantly at age 65, leading to low back pain and radiculopathy. Paraspinal muscle biopsy showed morphologic evidence of a mitochondrial myopathy. Diagnostic deficiencies of electron transport chain enzymes were not detected using standard bioassays, but mitochondrial immunofluorescence demonstrated many muscle fibers totally or partially deficient for complexes I, III, IV-I, and IV-IV. Massively parallel sequencing of paraspinal muscle mtDNA detected multiple deletions as well as a 40.9% heteroplasmic novel m.12293G > A (MT-TL2) variant, which changes a G:C pairing to an A:C mispairing in the anticodon stem of tRNA Leu^CUN^. Interestingly, these mitochondrial abnormalities were not detected in the blood of either the patient or her son, suggesting that the patient’s rapidly progressive late onset scoliosis was due to the acquired paraspinal mitochondrial myopathy; the cause of non-progressive scoliosis in the other two family members currently remains unexplained. Notably, this case illustrates that isolated mitochondrial myopathy can underlie rapidly-progressive adult-onset scoliosis and should be considered in the differential diagnosis of the primary axial myopathy.

**Electronic supplementary material:**

The online version of this article (doi:10.1186/s40478-014-0137-3) contains supplementary material, which is available to authorized users.

## Introduction

While adult scoliosis is a common disorder of the spine that often follows adolescent deformity, rapid progression of scoliosis with decompensation of posture occurs only rarely in the adult. Rapidly progressive adult-onset scoliosis is a debilitating and frequently painful condition that is poorly understood and typically considered idiopathic. One particularly well-known manifestation of adult-onset scoliosis is camptocormia or “bent-spine syndrome,” in which the patient’s spine is involuntarily flexed when standing or sitting [1]. Until recently, little was known about the heritable and acquired factors leading to camptocormia, aside from an unclear but reproducible relationship to Parkinson’s disease [2]–[4]. However, recent work has demonstrated that camptocormia can be due to a localized axial myopathy leading to spinal instability [5].

While axial myopathies can be secondary to other neuromuscular disorders, in some cases they appear to arise *de novo* and are designated primary axial myopathies. While etiologically heterogeneous, this group of conditions is clinically characterized by progressive paraspinal muscle weakness, myogenic pattern on EMG, and normal to mildly elevated creatinine kinase (CK) levels. Pathologically, affected paraspinal muscles typically show chronic myopathic changes reminiscent of muscular dystrophy (fiber size variation, fatty infiltration, and fibrosis [5]). Interestingly, mitochondrial changes (ragged red and cytochrome c oxidase (COX)-negative fibers) have also been observed in primary axial myopathies [6],[7], but are generally thought to represent non-specific age-related mitochondrial pathology. However, a recent illustrative report demonstrated that heritable mitochondrial DNA (mtDNA) mutations can also cause an axial myopathy [8]: a familial late-onset axial myopathy due to mutation in the mitochondrial tRNA Phe gene was demonstrated in two elderly sisters with a history of encephalopathy and ataxia.

Here, we report a case of rapidly progressive adult-onset scoliosis due to late-onset axial myopathy associated with multiple somatic mtDNA abnormalities but without chronic myopathic features typically observed in primary axial myopathies. This case demonstrates that adult spinal deformity may be secondary to a previously undiagnosed neuromuscular pathology and that primary axial myopathy can be due to an apparently isolated mitochondrial myopathy.

## Materials and methods

Biopsy of T8 paraspinal muscle was obtained intraoperatively during stage one of the two-stage spinal fusion. The flash-frozen muscle tissue was evaluated using a standard panel of muscle stains (hematoxylin and eosin [H&E]; modified trichrome; ATPase, pH = 9.4; NADH reductase; SDH; and COX). Light microscopy images were acquired with a DP72 digital camera on a BX41 bright-field light microscope using cellSens Entry 1.4 software (all by Olympus Corp.). For electron microscopy, ultrathin (80 nm) sections of the glutaraldehyde-fixed, Epon-embedded tissue were stained with 2% uranyl acetate. Sections were examined in a Tecnai G212 transmission electron microscope at 80 kV, with images obtained with a Hammamatsu camera model Orca HR.

Standard biochemical assays for enzyme activities of the electron transport chain were performed in duplicate on fresh frozen tissue at 30°C, according to previously published methods [9],[10]. The activities of complex I (NADH: ferricyanide dehydrogenase), complex II (succinate dehydrogenase), complex I + III (NADH: cytochrome C oxidase), complex II + III (succinate:cytochrome c reductase), and complex IV (cytochrome c oxidase) were measured using different electron acceptors/donors. Complex I was followed using the oxidation of NADH at 340 nm. Complex II activity was measured via the reduction of 2,6-dichloroindophenol (DCIP) at 600 nm. Citrate synthase was used as a marker for overall mitochondrial content.

Immunofluorescence staining with antibodies specific for subunits of five respiratory chain complexes was performed at the Molecular Neuropathology Laboratory, Texas Children’s Hospital. All antibodies were obtained from Abcam [Complex I, anti-NDUFS3 (ab110246); Complex II, anti-SDHB (ab14714); Complex III, anti-UQCRC2 (ab14745); Complex IV-I, anti-MTCO-1 (ab14705); Complex IV-IV, anti-COX IV (ab14744); Complex V, anti-OSCP (ab110276); PDH, anti-Pyruvate dehydrogenase E1 component subunit alpha (ab 110334)]. Cryosections of skeletal muscle were processed for immunofluorescence using a previously described protocol [11] with the above antibodies to respiratory chain enzyme complex proteins (green fluorescence); antibody to porin (Abcam ab14734; red fluorescence) served as reference control for mitochondrial density and distribution. Images were captured using Leica imaging system with Cytovision software. For each complex, fibers with strong red (porin) staining but no green staining were considered totally deficient (i.e. homoplasmic for mutant mitochondria), fibers with a patchy green and red staining were considered partially deficient (i.e. heteroplasmic for mutant mitochondria), while fibers lacking both red and green staining were considered mitochondrially hypodense.

Comprehensive mtDNA analysis by massively parallel (“next generation”) sequencing was performed on the paraspinal muscle and blood according to previously published methods [12],[13]. Briefly, the entire mitochondrial genome was amplified using long-range PCR. The PCR products were sequenced to approximately 20,000 depth of coverage using the Illumina Hiseq. 2000, with the Revised Cambridge Reference Sequence (rCRS NC_012920) used as reference. The quantitative measurements of reference base and observed base for a single variant were reported as mean percentage value, with coding region variants reported for heteroplasmy ≥1.5%. Sanger sequencing was performed for sequence confirmation of heteroplasmic findings in the coding region that exceeded 20% heteroplasmy. Massively parallel sequencing of mtDNA followed by Sanger sequencing of the breakpoint (deletion/junction) regions was used for mtDNA deletion analysis, as described previously [12],[13]. mtDNA copy number was determined according to published procedures [14]. Finally, nuclear genome regions that include 14 nuclear genes (C10ORF2, DGUOK, MPV17, OPA1, OPA3, POLG, POLG2, RRM2B, SLC25A4, SUCLG1, SUCLG2, TK2, and TYMP) known to be involved in the maintenance of mtDNA integrity and the deoxynucleotide salvage pathway were also analyzed using massively parallel sequencing.

## Results

### Clinical presentation

At age 65, the female patient developed progressive scoliosis consisting of decompensated deformity with severe sagittal and coronal plane imbalance that worsened rapidly over the next four years; past medical history was significant for stable scoliosis identified in the late adolescence. The patient also had a history of borderline hypertension and hyperlipidemia with transient ischemic attack (TIA) at age 62; rhytidectomy at age 64; and elbow surgery at age 66. Family history was positive for stable scoliosis in both her mother (who died at age 95) and her son (currently age 37). The patient reported increasing lumbosacral and leg pain as the scoliosis progressed; in addition, she developed mild dysphagia in the year preceding surgery, but has not undergone any additional testing to evaluate this symptom. She has normal stature (164 cm) and has reported no headaches, no myoclonus, no diplopia, no hearing loss, and no exercise intolerance.

**Figure 1:**
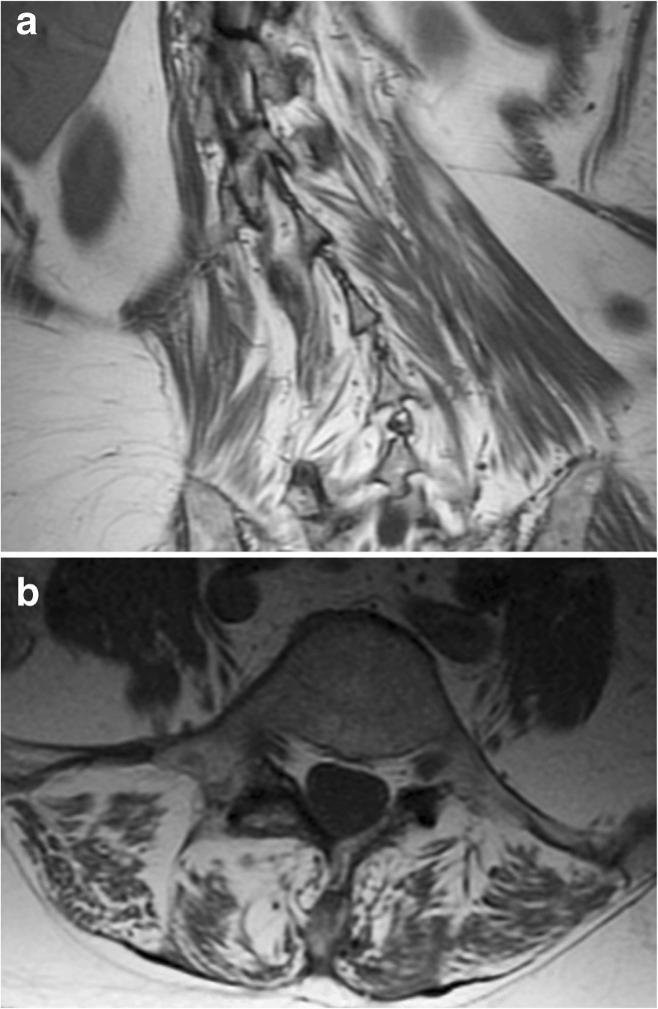
**Paraspinal muscle imaging.** Fatty replacement of the patient’s paraspinal musculature is demonstrated on coronal T1-weighted MRI image **(a)**, which also shows severe scoliosis, and on axial T2-weighted MRI image at spinal level L4 **(b)**.

Physical exam demonstrated decompensated spinal deformity with severe sagittal and coronal plane imbalance. There was no evidence of muscle wasting. Neurologic exam showed no evidence of ocular movement abnormalities, but mild left deltoid (4+/5) and neck flexion (4/5) weakness was present. X-ray imaging showed right-sided major thoracolumbar curve, with a progressive curvature from T10 to L4 (71 degrees with significant rotational element), along with flexional curvature at L4-5 to L5-S1 level. Magnetic Resonance Imaging (MRI) showed severe right curvature of the thoracolumbar spine (Figure 1a) with bilateral fatty atrophy of the posterior paraspinal musculature (Figure 1b; a somewhat nonspecific finding in the patent’s age group). There were no areas of severe stenosis and no evidence of intraspinal abnormalities. The patient had a normal CK level; electromyography of paraspinal muscles was not performed. At age 69, due to continued pain and deformity, the patient underwent a two-stage spinal fusion with paraspinal muscle biopsy. 20 months after completing both stages of surgery, the patient shows no evidence of recurrent deformity and has not developed additional neuromuscular symptoms.

### Muscle pathology and molecular genetics

**Figure 2:**
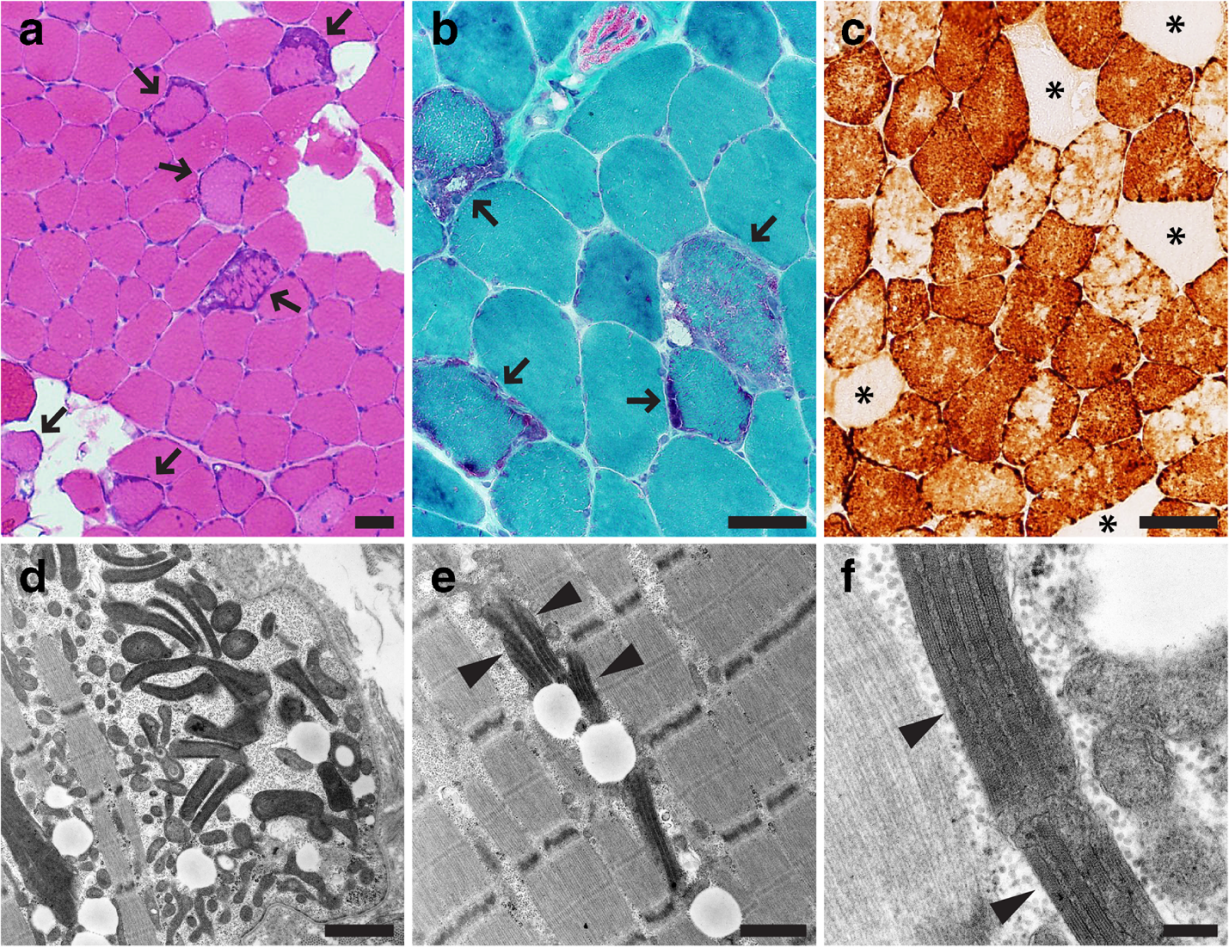
**Paraspinal muscle pathology.** Histochemical and ultrastructural analysis of T8 paraspinal muscle demonstrated evidence of a mitochondrial myopathy. **(a)** H&E stain (arrows indicate fibers with increase in basophilic staining indicative of mitochondrial hyperplasia), **(b)** modified trichrome stain (arrows indicate ragged red fibers), **(c)** COX (cytochrome c oxidase) stain (asterisks mark COX-negative fibers). Electron microscopy **(d-f)** shows mitochondrial hyperplasia, variation in the mitochondrial shape and size, and mitochondria with crystalline arrays (arrowheads). Of note, the crystalline arrays present in this case do not have a common “parking lot” appearance. Scale bars: **a-c**, 50 µm; **d**, 1.5 µm; **e**, 1 µm; **f**, 200 nm.

Light microscopic examination of the paraspinal muscle biopsy showed evidence of a mitochondrial myopathy, with frequent well-developed ragged red fibers demonstrated on trichrome stain and subsarcolemmal crescents (mitochondrial aggregates) on NADH and SDH stains; a significant proportion (approximately 6%) of muscle fibers was COX-negative (Figures 2a-c). [While relatively little is known about the baseline staining parameters of infrequently biopsied paraspinal muscles, mitochondrial abnormalities are uncommon: a search of the UCSF archival material uncovered four paraspinal muscle biopsies from the patients in the same age range (58–71 years) that showed a spectrum of myopathic and neurogenic changes but only rare ragged red fibers (maximally 1–2 per biopsy) and no COX-negative fibers.] H&E stain of the patient’s biopsy showed scattered degenerating/regenerating fibers and mild fiber size variation without evidence of inflammation or fibrosis. Ultrastructural analysis demonstrated mitochondrial hyperplasia and hypertrophy with frequent abnormal mitochondria and abundant mitochondrial inclusions (abundant matrical dense granules and well developed crystalline arrays; Figures 2d-f). Additionally, there was an increase in the number and size of lipid droplets and a mild increase in the amount of glycogen. Focal findings included disruption of the myofibrillary matrix, Z-band streaming, and a single focus of nemaline rods.

**Figure 3:**
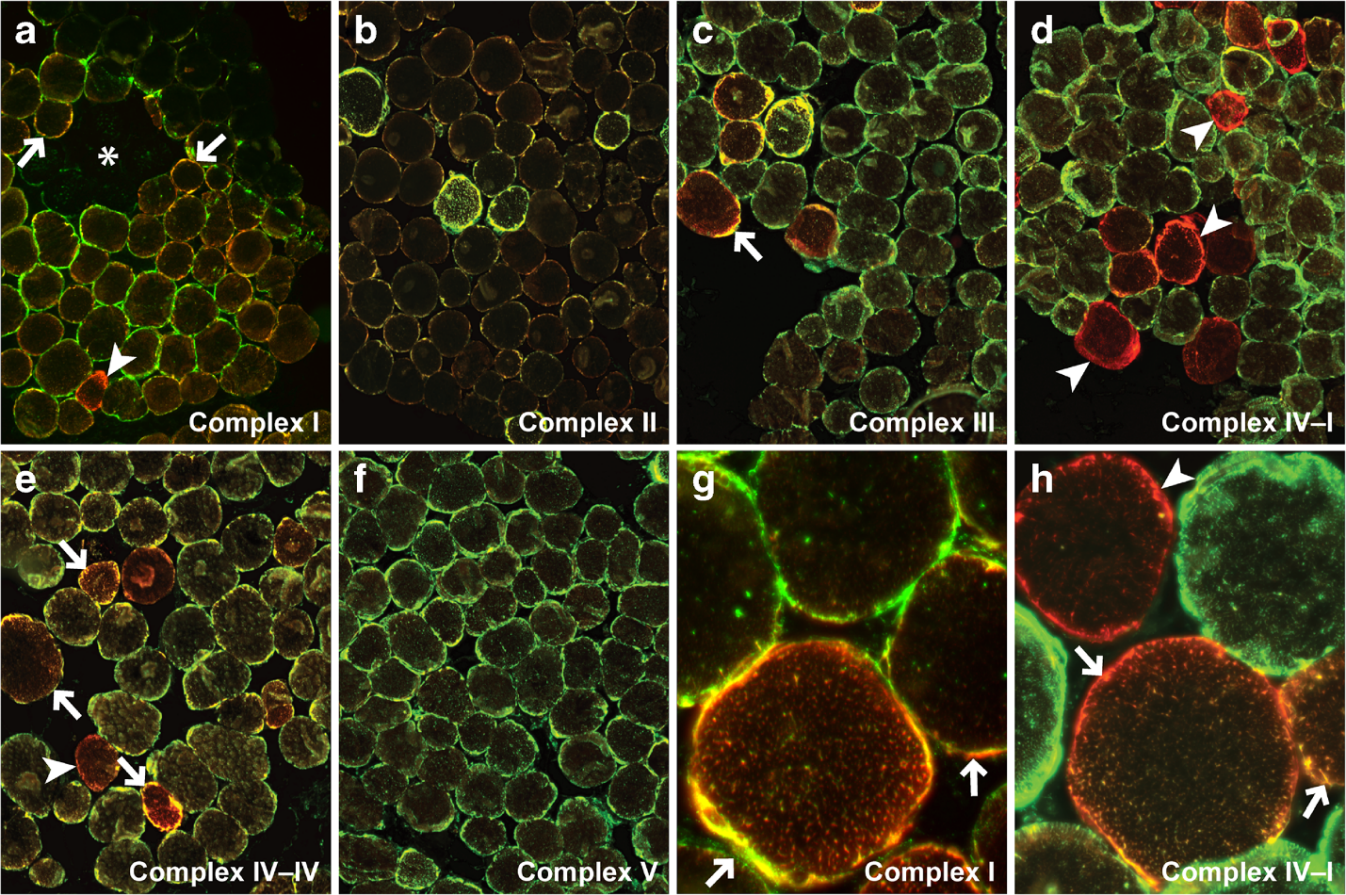
**Mitochondrial respiratory chain immunofluorescence.** Immunofluorescence staining (green) for Complex I (30 kD NDUFS3) [**(a)** and **(g)**], Complex II (30 kD FeS protein) **(b)**, Complex III (core 2) **(c)**, Complex IV-I (IV subunit I) **(d)**, Complex IV-IV (IV subunit IV) [**(e)** and **(h)**], and Complex V (OSCP) **(f)**; immunofluorescence staining for porin (red) is a reference control for mitochondrial density and distribution. Arrows, examples of partially deficient fibers; arrowheads, examples of totally deficient fibers; asterisk, an example of the area with fibers showing mitochondrial hypodensity. Original magnification: **a-f**, 100x; **g-h**, 400x.

Significant deficiencies of electron transport chain enzymes were not detected using standard biochemical assays (Table 1). Despite the absence of detectable biochemical abnormalities, immunofluorescence staining with antibodies specific for subunits of the five respiratory chain complexes showed many muscle fibers with reduced expression or total absence of individual complexes (Figure 3), presumably reflecting complex instability in the absence of mtDNA-encoded subunits. Complex I was the most prominently affected and showed widespread (>75%) reduced expression with many totally or partially deficient fibers, while other complexes showed milder abnormalities; the results are summarized in Table 2.

**Table 1:** **Mitochondrial respiratory chain enzyme analysis**

Complex	Electron transport chain activity assay	Patient (nmol/min/mg)	Patient (normalized values)	Control (+/– SD) (nmol/min/mg)^1^
I	NADH : ferricyanide dehydrogenase	548	154%	280 +/– 91
I + III	NADH : cytochrome C reductase (total)	34.2	86%	28.2 +/– 4.3
	NADH : cytochrome C reductase (rotenone sensitive)	5.3	46%	9.1 +/– 2.5
II	Succinate dehydrogenase	12.5	122%	8.1 +/–2.44
II + III	Succinate : cytochrome C reductase	3.19	51%	4.9 +/– 1.1
IV	Sytochrome C oxidase	45.2	122%	29.2 +/– 9.1
None	Citrate synthase (mitochondrial content marker)	355	100%	280 +/– 95

^1^Control values for quadriceps femoris (control values for paraspinal muscle are not available).

**Table 2:** **Mitochondrial respiratory chain immunofluorescence analysis**

Complex	Antigen	Tissue area showing reduced staining^1^	Partially or totally deficient fibers^2^
I	NDUFS3^3^	>75%	Frequent
II	SDHB^3^	25%-75%	None
III	UQCRC2^3^	<25%	Frequent
IV-I	MTCO-1^4^	<25%	Frequent
IV-IV	COX IV^3^	25%-75%	Frequent
V	OSCP^3^	<25%	None
PDH	PDH E1α^3^	>75%	None

^1^“Reduced staining” included totally deficient fibers, partially deficient fibers, and fibers with hypodensity of mitochondria; the involved area was semi-quantified as variable focal (<25% of the tissue area), variable regional (25-75% of the tissue area) and widespread (>75% of the tissue area).

^2^For each complex, fibers with strong red (porin) staining but no green staining were considered totally deficient, fibers with patchy green and red staining were considered partially deficient, while fibers lacking both red and green staining were considered to show mitochondrial hypodensity. Frequent was defined as ≥ 10% of muscle fibers in the entire specimen.

^3^Nuclear DNA-encoded subunit.

^4^mtDNA-encoded subunit.

**Figure 4:**
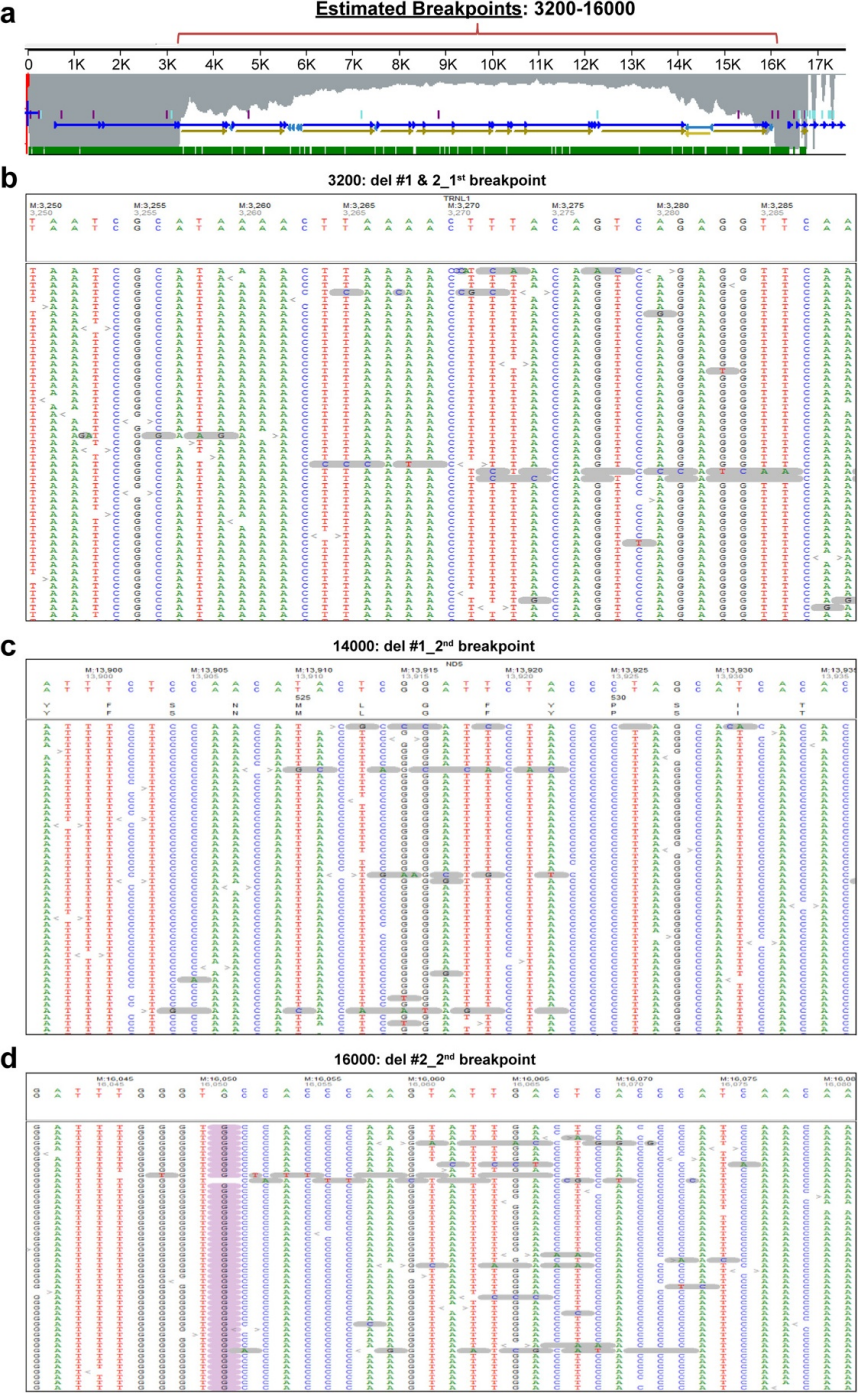
**Multiple mtDNA deletions are detected by next generation sequencing (NGS). (a)** NGS coverage profile suggests the presence of multiple mtDNA deletions, as described previously [12]. Further alignment of mismatched junction sequences identified multiple breakpoints; at least one possible 5´ deletion breakpoint **(b)** and two possible 3´ deletion breakpoints [**(c)** and **(d)**] were confirmed by Sanger sequencing (Additional file 1: Figure S1).

Comprehensive analysis of paraspinal muscle mtDNA by massively parallel sequencing detected multiple mtDNA deletions (Figure 4 and Additional file 1: Figure S1) and a novel 40.9% heteroplasmic m.12293G > A (MT-TL2) variant (Additional file 2: Figure S2), which changes a G:C pairing to an A:C mispairing in the anticodon stem of tRNA Leu. There was no evidence of mtDNA depletion (mtDNA copy number was 158% of tissue- and age-matched control) and no mutations were identified in 14 nuclear genes known to be involved in the maintenance of mtDNA integrity and the deoxynucleotide salvage pathway (C10ORF2, DGUOK, MPV17, OPA1, OPA3, POLG, POLG2, RRM2B, SLC25A4, SUCLG1, SUCLG2, TK2, and TYMP). Finally, no mtDNA abnormalities were detected in the blood of either the patient or her son, indicating that mtDNA abnormalities present in the paraspinal muscle were somatic in origin.

## Discussion

**Figure 5:**
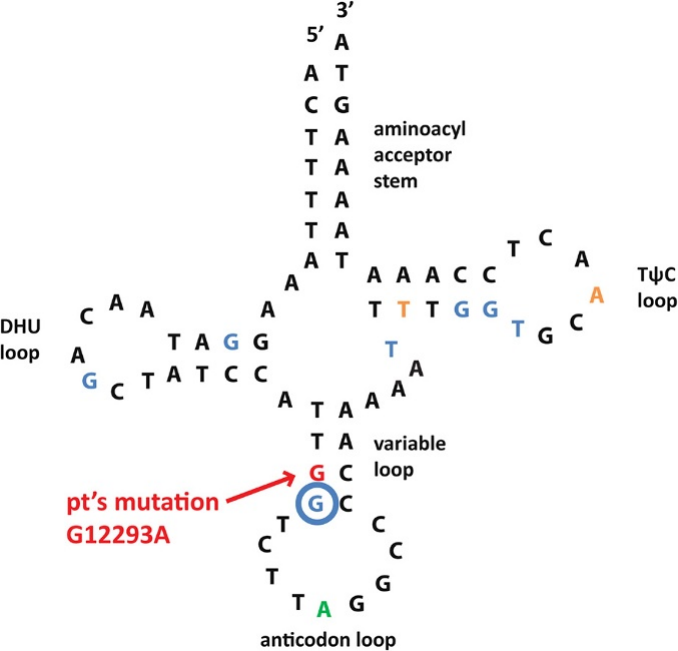
**Schematic diagram of MT-TL2.** Novel G12293A mutation is localized to the anticodon stem of the MT-TL2 (red arrow). Multiple point mutations in this tRNA exist, and have been linked to diseases including CPEO (residues in blue), MELAS (residue in green), and other mitochondrial myopathies (residues in yellow).

In the current report, we describe a patient with rapidly progressive adult-onset scoliosis (Figure 1) whose paraspinal muscle biopsy was diagnostic of a mitochondrial myopathy. Histochemical examination demonstrated numerous ragged red and COX-negative fibers, while ultrastructural analysis showed mitochondrial hyperplasia and hypertrophy with abundant abnormal mitochondria and frequent crystalline arrays (Figure 2). Although homogenized paraspinal muscle tissue did not show statistically significant biochemical deficits in electron transport chain activity assays (Table 1), mitochondrial immunofluorescence analysis revealed many totally and partially deficient fibers for complexes I, III, IV-I, and IV-IV (Table 2 and Figure 3). No deleterious mutations were found in fourteen nuclear genes known to influence mitochondrial DNA fidelity, but massively parallel sequencing of mtDNA identified multiple deletions (Figure 4 and Additional file 1: Figure S1) and a novel heteroplasmic (40.9%) variant in the MT-TL2 gene (Additional file 2: Figure S2), which changes guanine 12293 to adenine (m.12293G > A) (Figure 5).

What is the clinical significance of this novel mtDNA variant, which is not listed as a polymorphism in the Mitomap and mtDB mtDNA databases and was not detected in ~ 5000 individuals whose mtDNA was sequenced by the Baylor Medical Genetics Laboratories? The m.12293G > A mutation changes a G:C pairing to an A:C mispairing in the anticodon stem of MT-TL2 (tRNA Leu^CUN^); this position is conserved in all organisms examined by Weber *et al*. [15] and is adjacent to a similar mutation (m.12294G > A) that causes chronic progressive external ophthalmoplegia (CPEO), a classic mitochondrial disorder [16]. Several other point mutations in the MT-TL2 gene have been implicated in mitochondrial diseases, including CPEO, MELAS (mitochondrial myopathy, encephalopathy, lactic acidosis, and stroke syndrome), and other mitochondrial myopathies including isolated dilated cardiomyopathy [17]–[21],[15],[22],[16]. The absence of m.12293G > A (MT-TL2) variant in the patient’s blood suggests that this mtDNA variant is a novel point mutation that can lead to an isolated skeletal muscle (in this case paraspinal) myopathy. However, analysis of the patient’s paraspinal muscle mtDNA also showed multiple mtDNA deletions (Figure 4 and Additional file 1: Figure S1) that likely contributed to the mitochondrial myopathy phenotype; thus, studies of cybrids (immortalized cells whose mitochondria have been replaced by mitochondria with the point mutation of interest [23]) will be required to definitively demonstrate the pathogenicity of the newly identified MT-TL2 mutation.

Interestingly, the patient first developed scoliosis in the late adolescence, but her symptoms remained stable until age 65, when she experienced rapid progression. The family history of scoliosis (the patient’s mother and son also developed scoliosis in the mid-late adolescence) raised the possibility that a maternally-inherited mtDNA mutation was the underlying cause of their spine deformity, as was recently demonstrated for a family with a late-onset axial myopathy due to a mutation in the mitochondrial tRNA Phe gene [8]. However, no mtDNA abnormalities were identified in the patient’s son’s blood (to date, he has not undergone a muscle biopsy). The patient’s mother is deceased and her blood was not available for genetic testing; interestingly, however, her scoliosis remained stable throughout the whole adult life (> seven decades). Thus, it is likely that the patient’s initial scoliosis, which was relatively mild and remained stable for more than four decades, represents a separate familial disorder that still has an unexplained etiology; in contrast, the rapidly-progressive scoliosis the patient developed at the age of 65 is a consequence of the primary axial mitochondrial myopathy caused by the accumulation of somatic mtDNA abnormalities, including multiple mtDNA deletions and m.12293G > A mtDNA mutation. An alternative (but less likely) possibility is that m.12293G > A (MT-TL2) mutation is present in the patient’s blood and other tissues, but is below the current detection limit of the massively parallel sequencing (1% of heteroplasmy); in that scenario, the familial non-progressive scoliosis developed by all three family members would be due to m.12293G > A (MT-TL2) mutation, while the rapid symptom progression experienced only by our patient would be attributed to somatic acquisition of multiple mtDNA deletions. If the patient’s son undergoes a paraspinal muscle biopsy in the future, it may be possible to definitively differentiate between these two scenarios.

Despite obvious morphologic and genetic abnormalities present in the patient’s muscle biopsy, no significant deficiencies of electron transport chain enzymes were detected using standard biochemical assays (Table 1). In contrast, immunofluorescence assays for expression of five respiratory chain complexes showed widespread abnormalities (Figure 3 and Table 2). The discrepancy between the two methods could be due to a difference in sensitivity between imaging assays (which can distinguish affected from non-affected cells) and biochemical assays (which are performed on tissue homogenates). An additional methodological issue is the lack of proper enzyme activity control values for infrequently biopsied tissues (such as the paraspinal muscle): as in our case, a non-significant decrease in the complex I and IV activities was reported in a case of a familial axial myopathy associated with tRNA Phe mutation [8]. Finally, a problem with inter-laboratory reproducibility of mitochondrial respiratory chain biochemical assays has been reported [24],[25]. Thus, performing immunofluorescence in parallel with biochemical testing may increase the detection sensitivity for respiratory chain abnormalities, particularly when changes in the mitochondrial enzyme activity approach but do not meet the threshold for statistical significance.

In summary, this case illustrates that mitochondrial myopathy can underlie rapidly-progressive adult-onset scoliosis in the absence of significant chronic myopathic changes and that mitochondrial disorders should be considered in the differential diagnosis of the primary axial myopathy.

### Consent

Written informed consent was obtained from the patient for publication of this case report and any accompanying images. A copy of the written consent is available for review by the Editor-in-Chief of this journal.

## Additional files

## Electronic supplementary material

Additional file 1: Figure S1: Sanger sequencing confirms the presence of multiple mtDNA deletions. (a) PCR of the breakpoint region followed by Sanger sequencing confirmed the deletion at 3264-13923del10660 for the first 3´ deletion junction. (b) PCR of the breakpoint region followed by Sanger sequencing confirmed the deletion at 3264-16071del12808 for the second 3´ deletion junction. (TIFF 2 MB)

Additional file 2: Figure S2: Detection and quantification of the novel m.12293G > A mutation. (a) NGS sequence alignment shows the presence of m.12293G > A variant, which was present at 40.9% heteroplasmy in the paraspinal muscle. (b) The presence of m.12293G > A variant was confirmed by Sanger sequencing. (TIFF 1 MB)

Below are the links to the authors’ original submitted files for images.

Authors’ original file for figure 1

Authors’ original file for figure 2

Authors’ original file for figure 3

Authors’ original file for figure 4

Authors’ original file for figure 5
