# Supplementary figures and images for: Axial mitochondrial myopathy in a patient with rapidly progressive adult-onset scoliosis
Source: Acta Neuropathol Commun. 2014 Sep 16;2:137. doi: 10.1186/s40478-014-0137-3 (PMC4180433; doi:10.1186/s40478-014-0137-3)

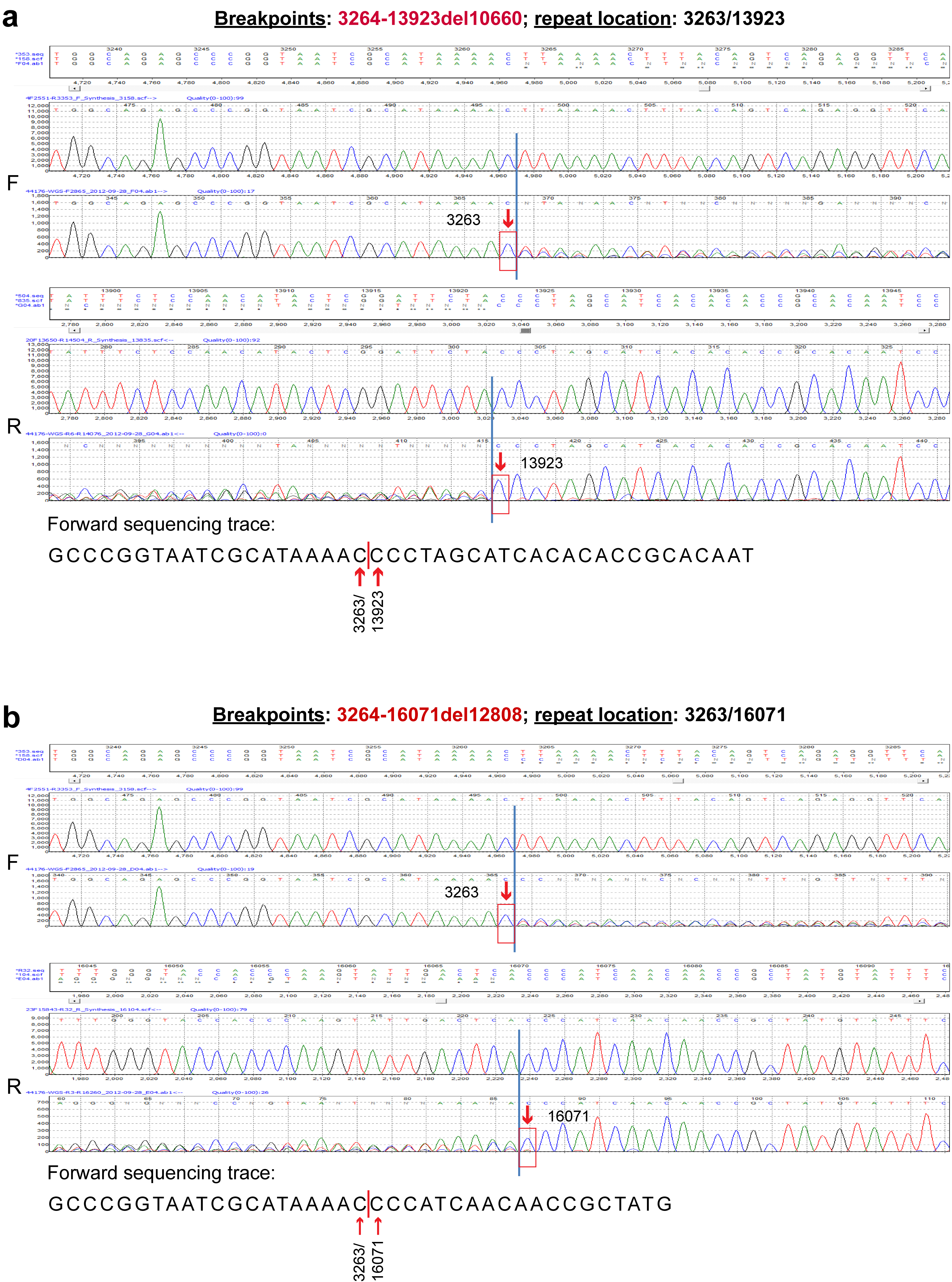

Supplement: Supplementary file 1 — Additional file 1: Figure S1: Sanger sequencing confirms the presence of multiple mtDNA deletions. (a) PCR of the breakpoint region followed by Sanger sequencing confirmed the deletion at 3264-13923del10660 for the first 3´ deletion junction. (b) PCR of the breakpoint region followed by Sanger sequencing confirmed the deletion at 3264-16071del12808 for the second 3´ deletion junction. (TIFF 2 MB) [file 40478_2014_9137_MOESM1_ESM.tiff]

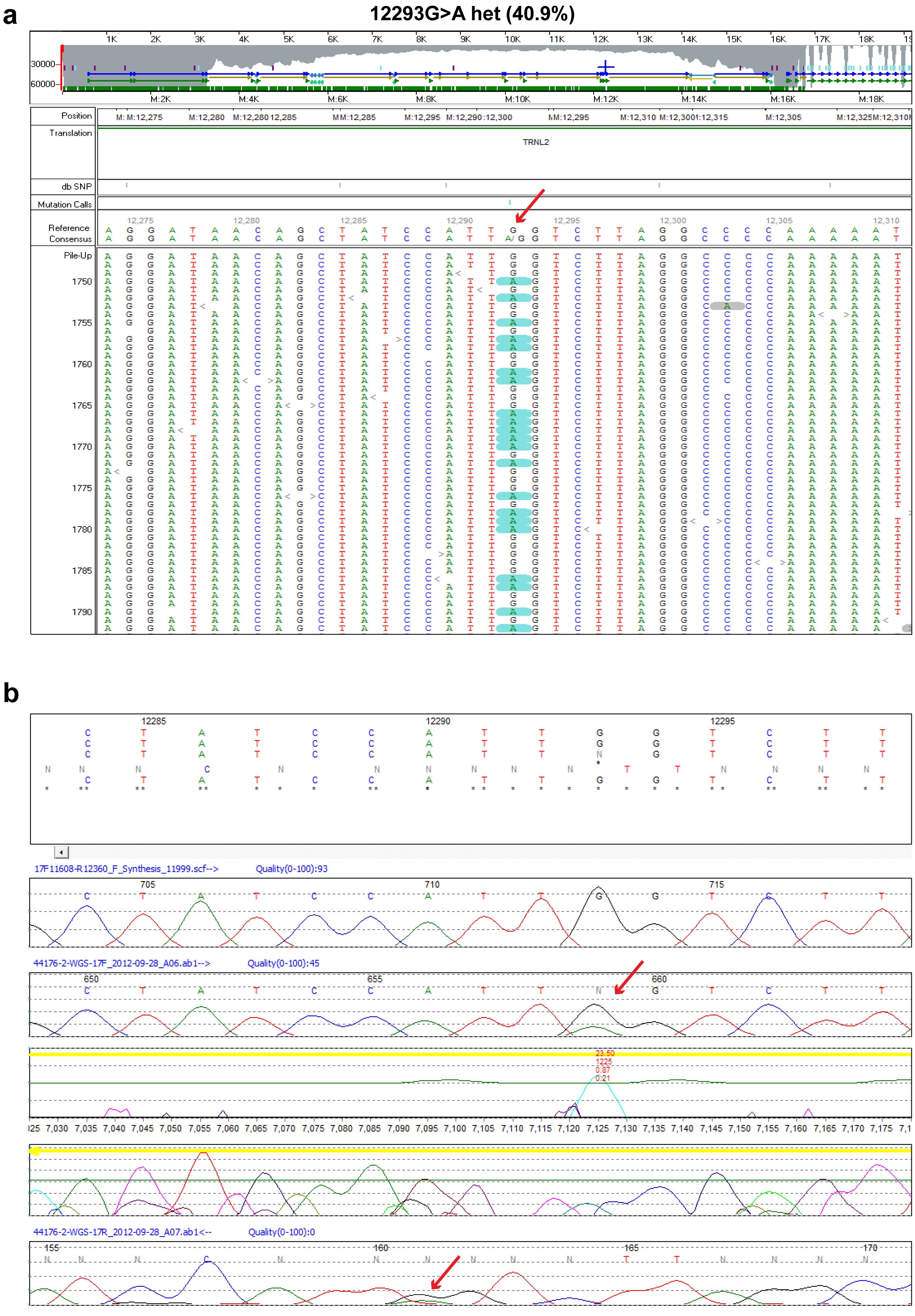

Supplement: Supplementary file 2 — Additional file 2: Figure S2: Detection and quantification of the novel m.12293G > A mutation. (a) NGS sequence alignment shows the presence of m.12293G > A variant, which was present at 40.9% heteroplasmy in the paraspinal muscle. (b) The presence of m.12293G > A variant was confirmed by Sanger sequencing. (TIFF 1 MB) [file 40478_2014_9137_MOESM2_ESM.tiff]

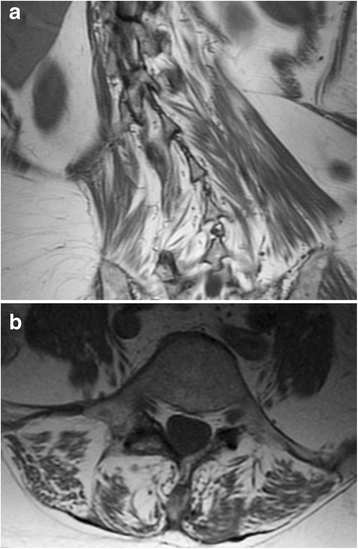

Supplement: Supplementary file 3 — Authors’ original file for figure 1 [file 40478_2014_9137_MOESM3_ESM.gif]

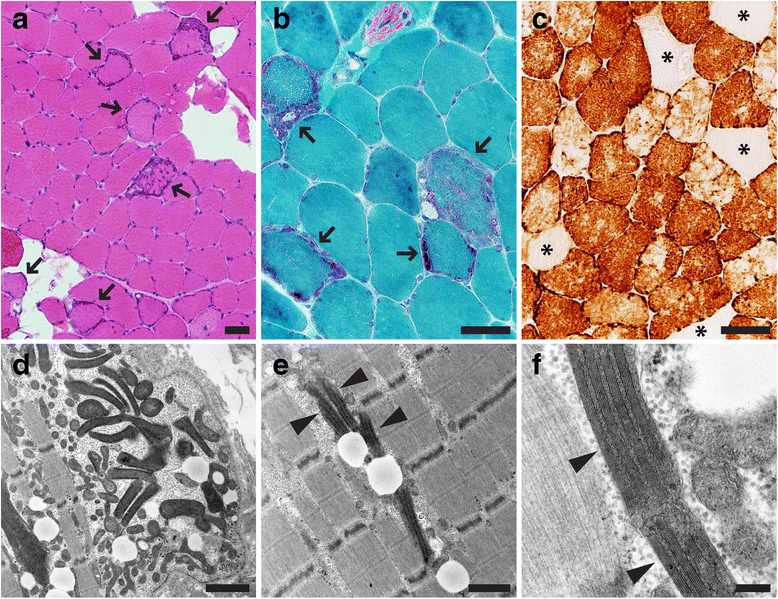

Supplement: Supplementary file 4 — Authors’ original file for figure 2 [file 40478_2014_9137_MOESM4_ESM.gif]

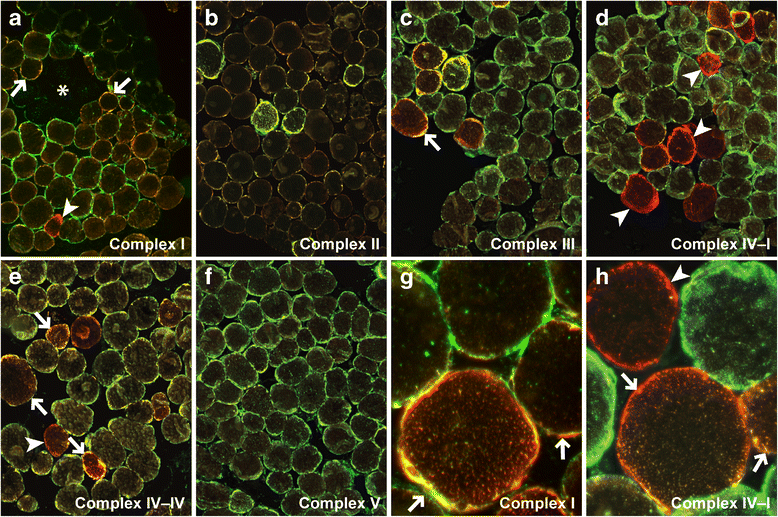

Supplement: Supplementary file 5 — Authors’ original file for figure 3 [file 40478_2014_9137_MOESM5_ESM.gif]

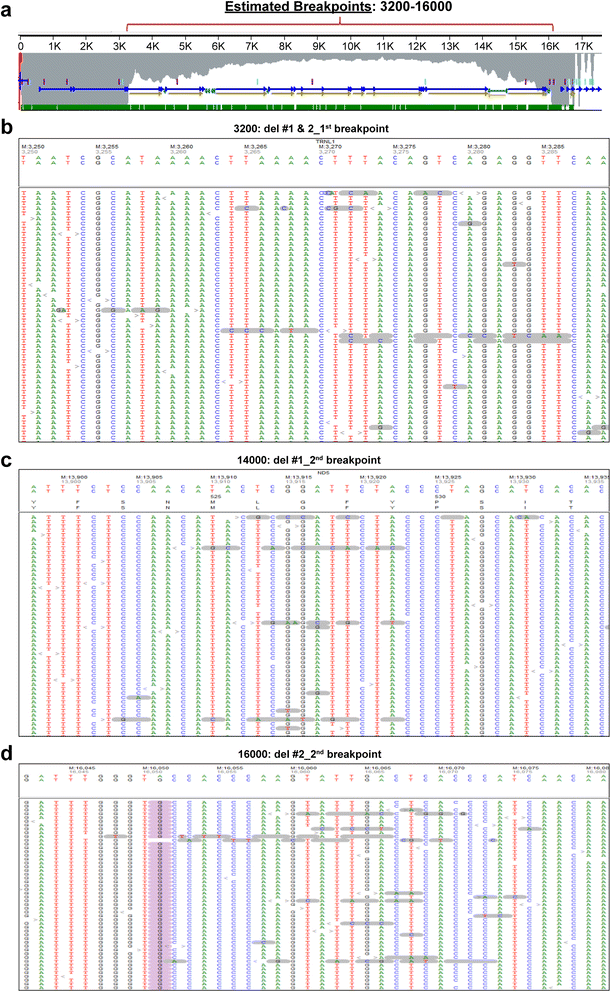

Supplement: Supplementary file 6 — Authors’ original file for figure 4 [file 40478_2014_9137_MOESM6_ESM.gif]

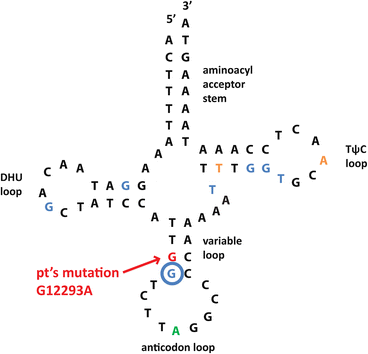

Supplement: Supplementary file 7 — Authors’ original file for figure 5 [file 40478_2014_9137_MOESM7_ESM.gif]
